# Clinical application of serum tumor abnormal protein in prostate cancer patients

**DOI:** 10.1186/s12885-024-12418-z

**Published:** 2024-05-31

**Authors:** Liusong Fu, Chi Zhang, Zewen Wang, Wei Tao, Jin Zhu, Yibin Zhou, Chuanyang Sun, Boxin Xue, Mengqi Yu, Lijun Xu, Yachen Zang

**Affiliations:** 1 Department of Urology, the Second Affiliated Hospital of Soochow University, Suzhou, China; 2 Department of Urology, Changzhou Maternal and Child Health Care Hospital, Changzhou, China

**Keywords:** Prostate cancer, Diagnosis, Tumor abnormal protein, Tumor marker

## Abstract

**Purpose:**

To explore the clinical value of tumor abnormal protein (TAP) in the diagnosis and prognosis evaluation of prostate cancer.

**Methods:**

This study enrolled a total of 265 patients who underwent prostate biopsy procedures from December 2017. TAP levels were assayed in their blood samples using a validated TAP testing kit. Comprehensive pathological assessments, including Gleason scores, TNM staging, and AJCC prognosis stages, were conducted on prostate cancer patients. Further analysis was carried out to examine the correlation between TAP expression levels and various clinical characteristics.

**Results:**

A significantly elevated TAP concentration was discerned in prostate cancer patients relative to those with benign prostate hyperplasia. Moreover, a significantly elevated TAP expression was detected in prostate cancer patients with high Gleason score (≥ 8) and advanced stages (III and IV), as compared to those with Gleason scores of 6 and 7 and lower stages (I and II). When diagnosing prostate cancer in gray area of PSA, TAP demonstrated superior diagnostic capabilities over PSA alone, with higher diagnostic sensitivity, specificity and accuracy than fPSA/tPSA ratio. Additionally, post-surgical or hormonal treatment, there was a marked reduction in TAP expression level among prostate cancer patients.

**Conclusion:**

The assessment of TAP presents itself as a promising tool for early diagnosis and holds potential for sensitivity in monitoring treatment reponse in prostate cancer patients.

## Introduction

Prostate cancer (PCA) is one of the most common malignancies in men. It is associated with age, genetics, diet, environment and sex hormones [1, 2]. According to its staging characteristics, early diagnosis and follow-up active treatment of prostate cancer patients is the key to improve the survival and prognosis of patients.

Currently, the diagnosis of prostate cancer predominantly encompasses digital rectal examination (DRE), prostate-specific antigen (PSA) and transrectal prostate biopsy under ultrasound guidance. Notably, the definitive diagnosis relies mainly on prostate biopsy coupled with histopathological evaluation [3]. Despite their diagnostic significance, invasive nature and potential complications render these biopsies less than ideal as a preliminary screening tool for routine use, thereby posing a challenge in clinical practice [4].

In comparison to other neoplastic diseases, prostate cancer exhibits a more pronounced degree of intra-tumor heterogeneity, a characteristic that has garnered more attention in recent years [5]. Consequently, there has been a growing trend towards the employment of multiple tumor markers for improved diagnostic precision. These detection methods offer relative convenience and repeatability, rendering it a potential tool for use as an indicator of early diagnosis, preliminary screening and continuous monitoring for tumor progression in patients. When it comes to the early diagnosis of PCA patients, PSA stands out as the most extensively utilized tumor marker. However several studies have evidenced that reliance solely a single PSA test lacks specificity and precision in accurately distinguishing between early-stage prostate cancer and benign prostatic hyperplasia. This limitation may lead to an increased likelihood of unnecessary biopsy procedures [6, 7].

Tumor abnormal protein (TAP), synonymous with aberrantly glycosylated glycoprotein, represents a class of anomalous glycosylation products shed during the metabolic processes of neoplastic cells. Its detection results indirectly indicate that the number and level of tumor cells are closely related to the occurrence, development and metastasis of tumors [8]. TAP becomes easily discernible in the peripheral blood upon reaching a detectable concentration threshold, thereby offering a minimally invasive and highly convenient diagnostic modality with minimal patient discomfort. Numberous studies have attested to diagnostic and/or prognostic significance of TAP measurements in a range of solid tumors, including gastric cancer, bladder cancer, and colorectal cancer [9–11]. Moreover, TAP holds considerable potential as a pan-tumoral marker with applicability to the diagnosis of pancreatic, gallbladder, bile duct, and liver cancers [12]. Despite this expanding repertoire of applications, the utility of TAP for prostate cancer diagnosis remains unexplored. The present study was designed with explicit objective of elucidating the diagnostic value and prognostic implications of serum TAP testing for prostate cancer.

## Materials and methods

### Clinical data

The present prospective study was initiated in December 2017 and encompassed a cohort of 265 patients who underwent prostate biopsy in the department of urology of the Second Affiliated Hospital of Soochow University. Among these patients, 135 were confirmed as prostate cancer, while the remaining 130 were diagnosed with benign prostatic hyperplasia (BPH). This study was completed with the informed consent of all the patients. Inclusion criteria for the study were: (1) The patients who underwent a prostate biopsy, with subsequent histopathology confirming a diagnosis of benign prostatic hyperplasia or prostate cancer. (2) The patients agreed to participate in this study. Exclusion criteria: (1) The patients were diagnosed with other malignancies. (2) Factors influencing PSA levels, e.g., acute prostatitis, 5α-reductase inhibitor use etc.3) Interfering factors for TAP assay, including active rheumatic disease, unhealed fractures, autoimmune disorders, tuberculosis, severe cardiac, pulmonary, hepatic, or renal impairment.

### Prostate biopsy

Prostate biopsies was performed in patients for the following indications: abnormal digital rectal examination, PSA elevation (> 4 ng/ml), or suspected prostate cancer based on MRI or other examinations. All the patients were performed transrectal ultrasound (TURS) guided biopsy, utilizing a standard 10 + X cores sampling protocol. The biopsy specimens were promptly fixed in neutral buffered formalin and subsequently dispatched for histopathological assessment.

### Detection of TAP

#### Reagent

TAP reagent utilized in this study was sourced from Zhejiang Ruisheng Medical Technology Limited [13]. The reagent contains agglutinin, which faciliates the aggregation of diverse aberrant glycan glycoproteins and calcium-histones into distinctive, crystal-like aggregates. These formed structures are amenable to visualization under a standard light microscope.

#### Blood collection, preparation and testing

Blood samples were obtained from the distal phalanx of the middle finger of all participating patients. The collected blood was evenly distributed onto three separate slides, promptly spread to create thin blood smears, and allowed to air-dry at ambient temperature for 10 min. Subsequently, the reagent was thoroughly homogenized before being applied to each slide, resulting in the formation of three distinct, homogeneous deposits. These prepared slides were then meticulously positioned within a designated purification chamber, maintained at a constant temperature of 25℃ and a relative humidity of 50%. Following an incubation period of two hours, the slides were examined to record the outcomes of the assay.

#### Determination of detection results

**Fig. 1:**
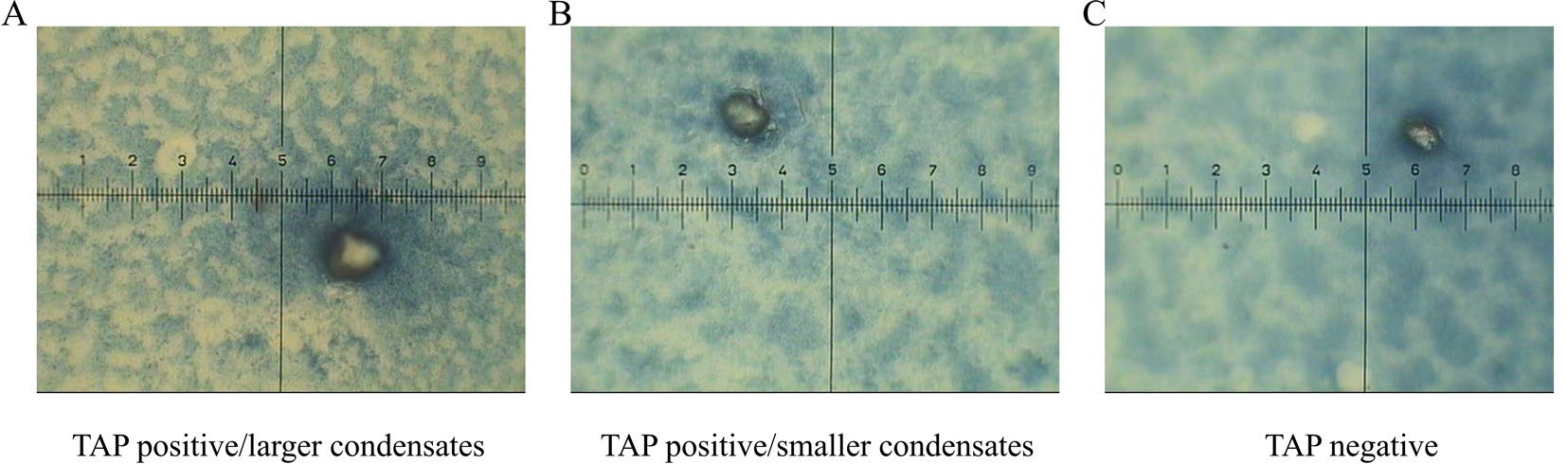
TAP detection results. **A** TAP positive/larger condensates, **B**. TAP positive/smaller condensates, **C**. TAP negative (magnification, ×400)

TAP positive/larger condensates: having a single condensate with an area of ≥ 225mm^2^ (Fig. 1A) or having 3 or more condensates with an area of 121 to 225mm^2^.

**Fig. 2:**
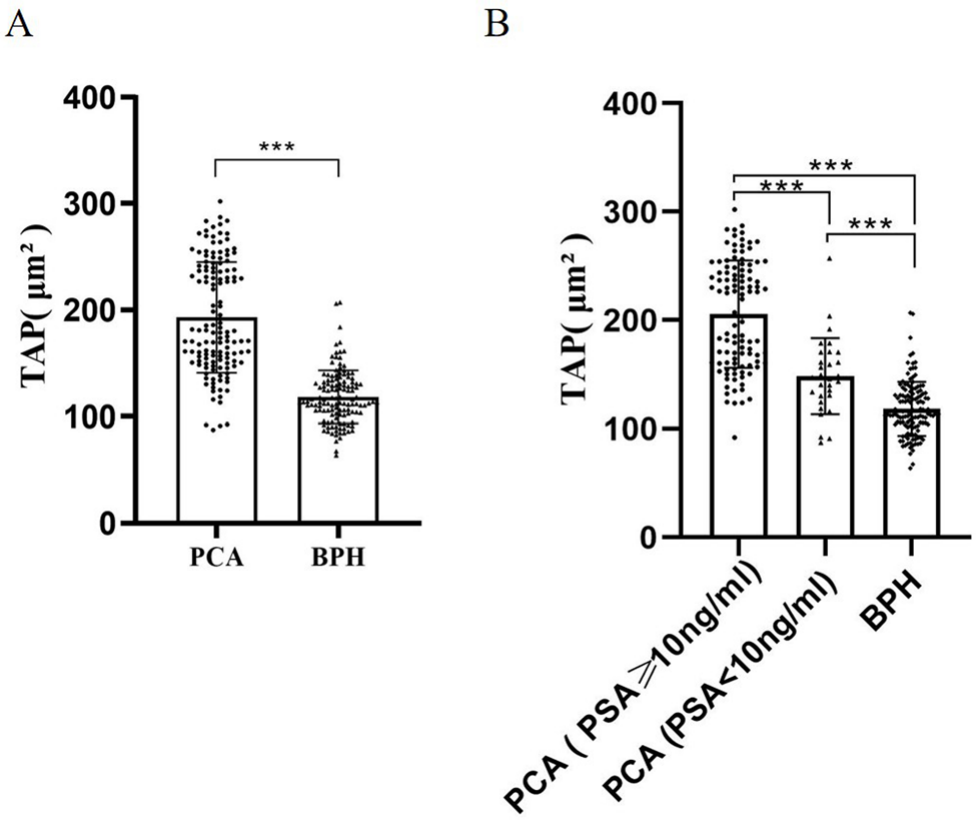
TAP expression is increased in prostate cancer. **A** Expression of TAP in prostate cancer and benign prostatic hyperplasia patients. **B** Expression of TAP in high-PSA expression. **C** PCA group (PSA > 10ng/ml), low-PSA expression PCA group (4 < PSA ≤ 10ng/ml) and BPH patients. (***P < 0.001, **P < 0.01)

TAP positive/smaller condensates: having 2 condensates with an area of 121 to 225mm^2^ (Fig. 1B) or having 3 or more condensates with an area of 81 to 121mm^2^. TAP negative: Samples were confirmed as TAP-negative when there was no condensate, or condensates with an area of < 81mm^2^ (Fig. 1C) or 2 or less condensates with an area of 81 to 121mm^2^ [14].

### Statistical analysis

Statistical analysis was performed using the Statistical Package for the Social Sciences (SPSS) version 20.0 (SPSS Inc., USA) and GraphPad Prism 8.0 (GraphPad software, USA). Measurement data were expressed as mean ± standard deviation, and comparison between groups was using Student’s t test. *P* < 0.05 was considered to indicate statistical significance.

## Results

### TAP expression is increased in prostate cancer

In the present study, the mean TAP condensate area was determined to be 193 ± 52.1 mm^2^ in a cohort of 135 prostate cancer patients, whereas for 130 BPH patients, the corresponding value was 118.3 ± 24.9 mm^2^. Concurrently, PSA levels averaged 167 ± 512.6 ng/ml and 11.47 ± 15.87 ng/ml in prostate cancer and BPH patients, respectively. Statistical analysis revealed a highly significant difference (*P* < *0.001*) between these two patient polulations (Fig. 2A). The detailed TAP expression profile and characteristics of the prostate cancer patients were presented in Table 1. Subsequently, all prostate cancer patients were stratified into high-PSA expression group (PSA > 10 ng/ml) and low-PSA expression group (4 < PSA ≤ 10 ng/ml). Our findings indicated significant differences in TAP expression levels among prostate cancer and BPH patients in these groups (*P* < *0.001*). Notably, the difference was more pronounced within the high PSA-expression group (Fig. 2B). Within the low-PSA cohort, prostate cancer patients and those with BPH exhibited mean PSA levels of 6.97 ± 1.56 ng/mL and 6.67 ± 1.56 ng/ml respectively, demonstrating no statistically significant difference. These data collectively indicate that TAP expression is increased in prostate cancer, and suggest that the assessment of TAP in peripheral blood holds comparable diagnostic value to PSA in the early screening of prostate cancer.

**Table 1:**
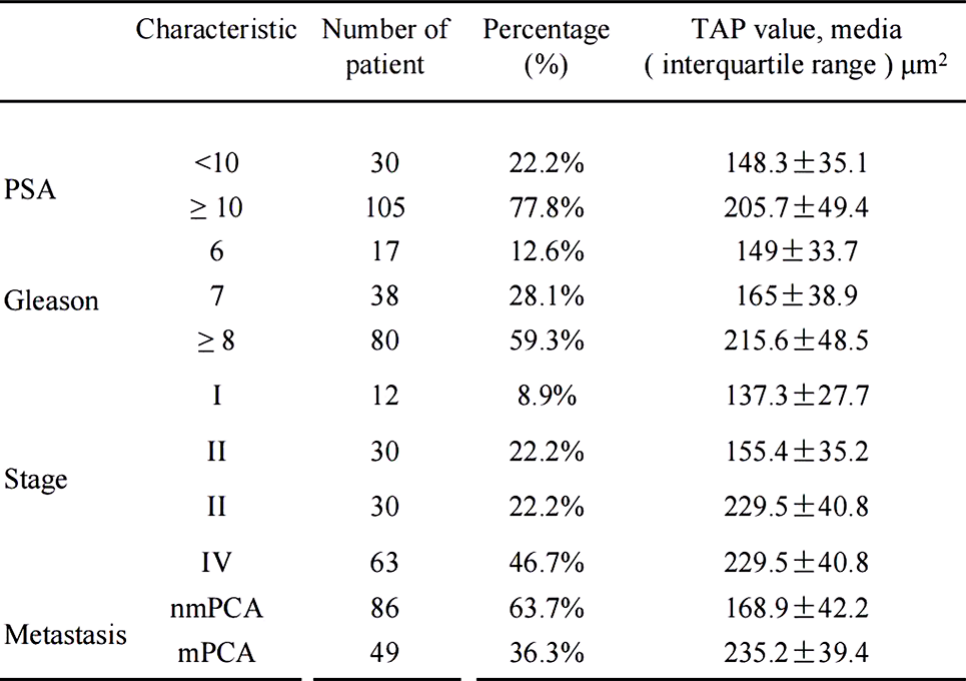
The TAP expression detail and characteristics of the prostate cancer patients

### TAP expression is increased in aggressive and metastasis prostate cancer

**Fig. 3:**
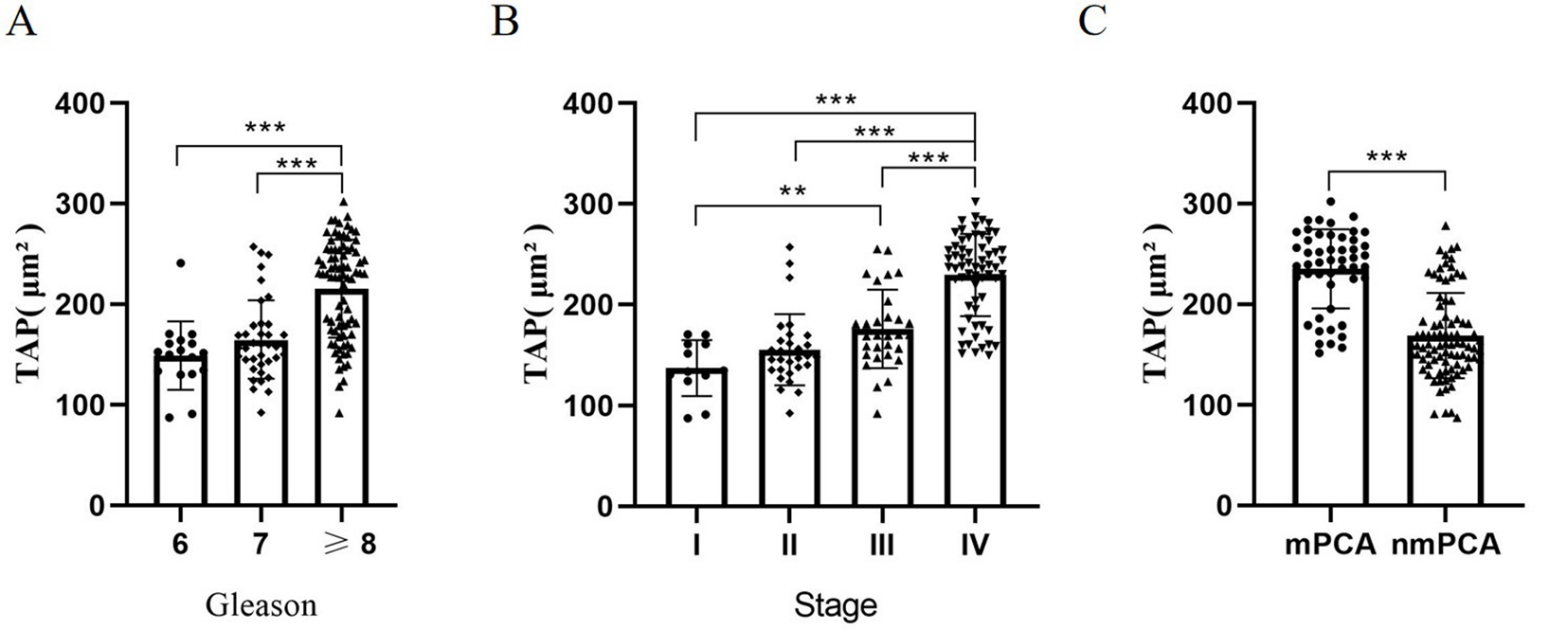
TAP expression is increased in aggressive and metastasis prostate cancer. **A** Expression of TAP in Gleason 6, 7 and ≥ 8 groups of prostate cancer patients. **B** Expression of TAP in PCA patients of stages from I to IV according to AJCC staging system. **C** Expression of TAP in non-metastasis and metastasis prostate cancer groups. (***P < *0.001*, **P < *0.01*)

To investigate the association between TAP expression and progression of prostate cancer, we conducted a comparative analysis of TAP levels across varying Gleason grades and clinical stages. Upon stratification of TAP expression according to Gleason scores of 6, 7 or ≥ 8, a statistically significant elevation in TAP expression was observed in Gleason score ≥ 8 group compared to both Gleason scores 6 and 7 (*P* < *0.001*). The expression of TAP in Gleason 7 group was comparable to that in Gleason 6 group (Fig. 3A).

Regarding TAP expression across all clinical stages of prostate cancer, the results are presented in Fig. 3B. The patients were categorized into stages I through IV according to AJCC staging system. A dramatically increased TAP expression was evident in advanced stages (III and IV) relative to early stages (I and II) (*P* < *0.001*). Moreover, the expression of TAP in stage IV was significantly higher than in the remaining three stages (*P* < *0.001*). These findings collectively indicated that the expression of TAP in prostate cancer increases appreciably with increasing Gleason grade and advancing clinical stage. Gleason grading serves as a crucial parameter for assessing the malignant potential of prostate cancer [15], while the AJCC clinical stage represents a standardized measure of the early and advanced stage of prostate cancer [16]. Hence, our data indicate a positive correlation between TAP expression and the overall malignancy of prostate cancer.

Furthermore, patients were divided into non-metastatic and metastatic prostate cancer groups, revealing a dramatically higher TAP expression in metastatic group compared to non-metastatic group (Fig. 3C). These results indicate that TAP expression was elevated with increasing tumor grade and advanced tumor stage in human prostate cancer, further solidifying its potential role as a biomarker of disease aggression and progression.

### TAP expression is associated with prostate cancer treatment

**Fig. 4:**
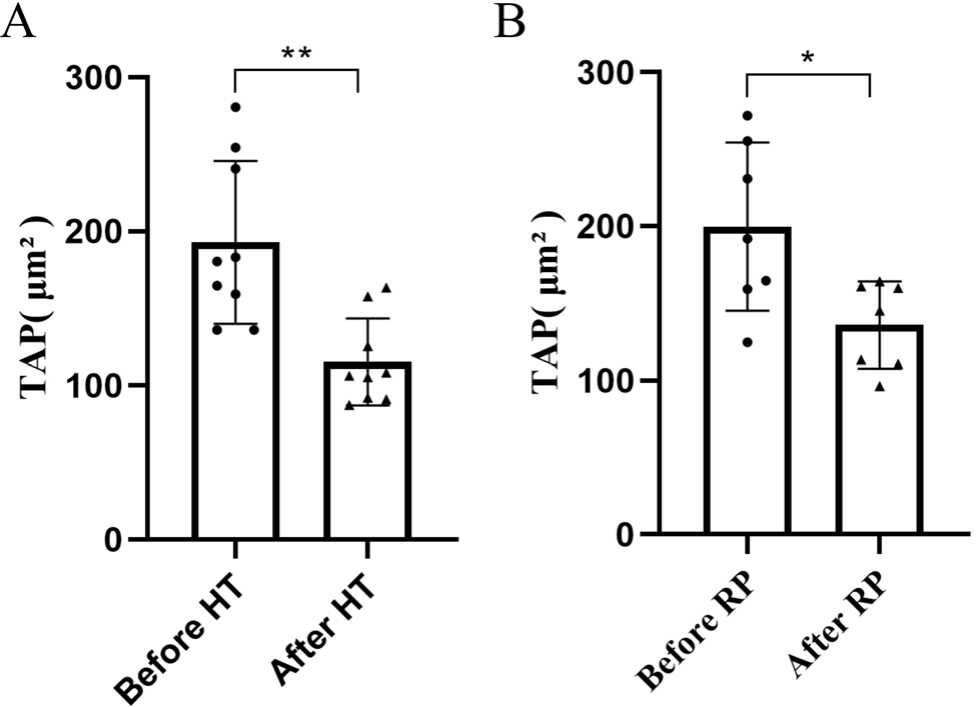
**A** TAP expression is associated with prostate cancer treatment. **B** TAP expression in prostate cancer patients pre-operation and 6 months post-operation of laparoscopic radical prostatectomy. TAP expression before and after prostate cancer therapy of ADT combine with bicalutamide or abiraterone. ( **P < 0.01, *P < *0.05*)

To explore the association between the expression of TAP and the therapeutic response in prostate cancer, we assessed TAP expression 6 months later post-laparoscopic radical prostatectomy in prostate cancer patients. As showed in Fig. 4A, a statistically significant reduction in TAP expression was observed in these patients following surgical intervention(*P* < *0.05*). Additionally, we also conducted examinations on TAP expression 6 months after initiation androgen deprivation therapy (ADT) combine with either bicalutamide or abiraterone in patients with advanced prostate cancer or those who were deemed intolerent to sugical entervention. It was showed that the TAP expression decreased following hormonal therapy(*P* < *0.01*) (Fig. 4B). These results suggest a positive correlation between TAP expression and the therapeutic efficacy of both radical ptostatectomy and hormonal therapies in protate cancer management. Consequently, TAP emerges as a promising candidate for monitoring the treatment response in these patients, potentially offering valuable insights into the effectiveness of therapeutic interventions and guiding future clinical decision-making.

### TAP has more diagnostic capabilities than other diagnostic methods in prostate cancer

**Fig. 5:**
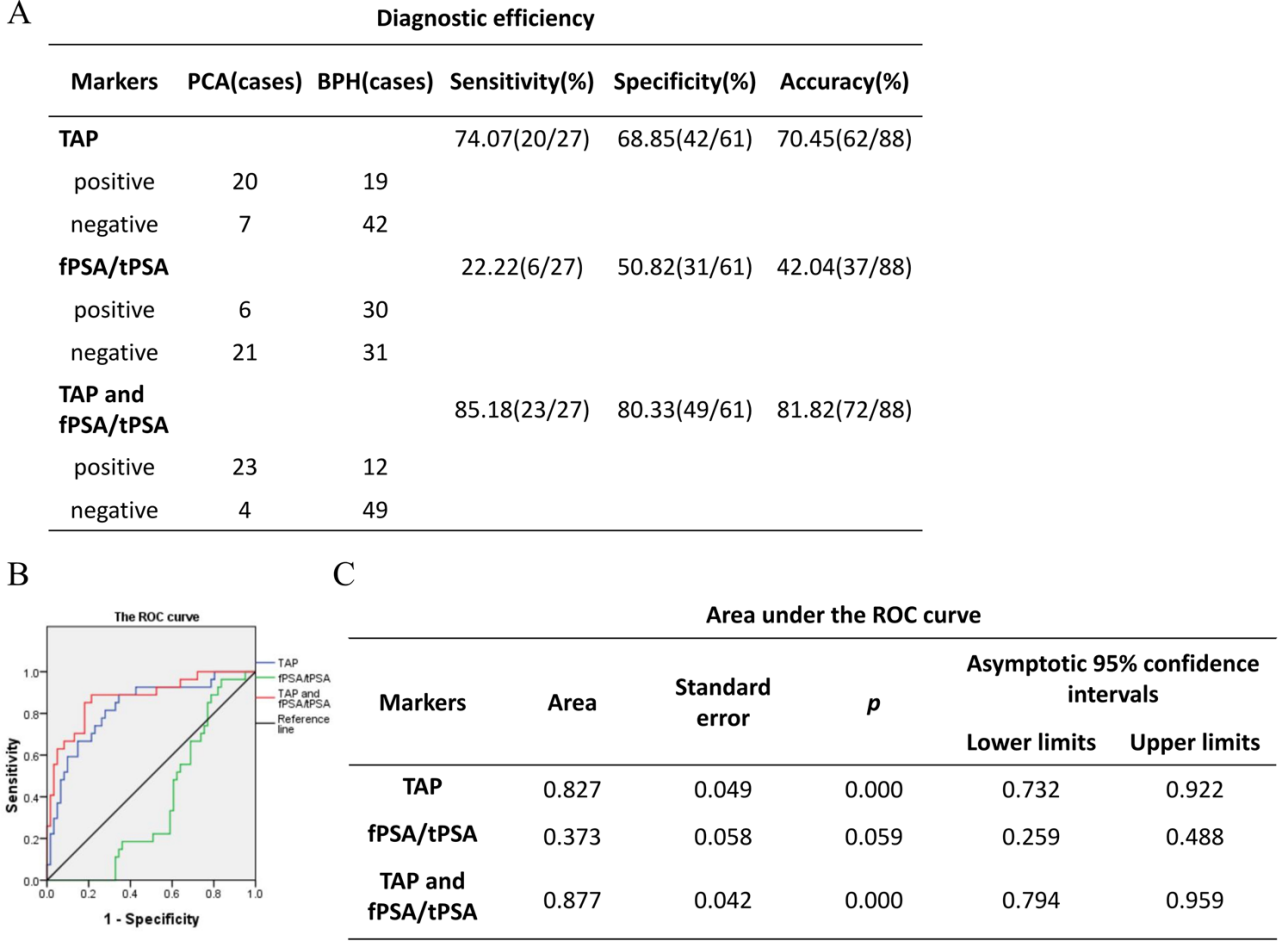
**A** TAP has more diagnostic capabilities than other diagnostic methods in prostate cancer The diagnostic efficacy of TAP, fPSA/tPSA ratio and combination of two indexes in patients with PSA gray area. **B** and **C**. ROC curves of TAP, fPSA/tPSA ratio and combination of two indexes in patients with PSA gray area

PSA constitutes the benchmark for prostate cancer diagnosis, yet it is accompanied by a diagnostic ambiguity. Specifically, the 4 ng/mL to 10 ng/mL PSA range presents a zone of uncertainty, often referred to as the “gray area”, wherein false-negative diagnoses of prostate cancer can occur [17]. With the aim of elucidating the diagnostic efficacy of TAP in this context, we focused our analysis on patients whose PSA values fell within this gray area. We found that the diagnostic sensitivity, specificity and accuracy of TAP in patients with gray area were 74.07%, 68.85% and 70.45%. By contrast, the fPSA/tPSA ratio exhibited lower performance, with sensitivity, specificity and accuracy of 22.22%, 50.82% and 42.04%. Intriguingly, the concurrent assessment of both TAP and fPSA/tPSA ratios led to a substantial improvement in diagnostic prowess, achieving respective sensitivity, specificity and accuracy of 85.18%, 80.33% and 81.82% (Fig. 5A). Thus, TAP outperformed the fPSA/tPSA ratio in terms of diagnostic sensitivity, specificity and accuracy in the PSA gray area, while the combined evaluation of these two indices surpassed the diagnostic value of either index individually.

Subsequently, we constructed receiver operating characteristic (ROC) curves to further assess the discriminatory power of TAP and PSA in the context of prostate cancer diagnosis within the gray area. The area under the ROC curve (AUC) for TAP was calculated as 0.827, indicating a statistically significant difference(*P* = 0.000). Conversely, the AUC for PSA alone was significantly lower, at 0.373 (Fig. 5B and C). These results demonstrate that TAP possesses superior diagnostic capabilities compared to PSA for prostate cancer detection within the gray area. Notably, the combined diagnostic efficacy of TAP and PSA was even more pronounced, yielding an AUC of 0.877. This highlights the potential for enhanced diagnostic accuracy when integrating TAP and PSA ratio measurements in the assessment of prostate cancer within the challenging PSA gray area.

## Discussion

TAP, an abnormal glycosylated protein emanating from the metabolic processes of tumor cells, has been demonstrated in previous studies to serve as an indirect indicator of tumor occurrence, progression and metastatic potential [18]. In gastric cancer patients, TAP levels have been shown to be significantly elevated compared to those in healthy controls, with a strong association observed between heightened TAP expression and longer progression-free survival among afflicted patients [19]. Integrating TAP examination alongside clinical manifestations and symptoms, it has proven valuable in the diagnostic workup of urothelial carcinoma of the bladder [10]. In colorectal cancer patients, the detection of TAP has consistently exhibited high degrees of both sensitivity and specificity [11]. In breast cancer, the concurrent measurement of TAP in conjunction with three conventional three conventional serum biomarkers has been found to yield the highest sensitivity and specificity for the diagnosis of breast cancer [13].

In the present study, we observed significantly elevated TAP expression in prostate cancer patients compared to those with benign prostate hyperplasia. This finding was consistent with previous studies of other malignancies that the positive rate of TAP in selected patients with cancer is significantly higher than that of nontumor patients [12]. Additionally, we demonstrated a positive correlation between TAP expression and the well-established prostate cancer marker PSA, confirming TAP’s potential role as a promising diagnostic indicator. Further statistical analysis revealed that TAP exhibits commendable sensitivity and specificity for prostate cancer diagnosis, as evidenced by a wide variety of indicators, including omission diagnostic rate, mistake diagnostic rate, Youden index, positive predictive value and negative predictive value. Moreover, tt was suggested that combining TAP with established markers and and adopting a holistic diagnostic approach could enhance the precision of tumor auxiliary diagnosis.

Furthermore, we revealed a progressive increase in the positive rate of TAP expression concomitant with advancement of prostate cancer, leading support to its potential utility in early disease detection. By categorizing patients according to AJCC staging system, ranging from I to IV, a marked increased TAP expression was evident in advanced stage (III and IV) ompared to early stage (I and II). Notably, the expression of TAP in stage IV patients was significantly higher than in those at stage I, II and III. The Gleason grading system, a critical prognostic factor in prostate cancer, plays a pivotal role in stratifying patients into risk categories and guiding therapeutic decision-making. When TAP expression was stratified according to Gleason score, a statistically significant elevation of TAP expression was observed in Gleason score ≥ 8 group relative to those with Gleason scores of 6 and 7. These findings strongly suggest an association between TAP expression and aggressive prostate cancer, implicating TAP as a potential biomarker for identifying high-risk tumors.

PSA is a widely employed serum biomarker for prostate cancer detection and surveillance, significantly contributing to early disease recognition. However, PSA testing is not without limitations, particularly in the context of a diagnostic gray area that exists within the range of 4 ng/mL to 10 ng/mL. Although an elevated PSA level above the traditional threshold of 4 ng/mL may prompt further evaluation for prostate cancer, the gray zone reveals a marked reduction in the specificity of the test. Many men with PSA levels within this range do not have prostate cancer but rather benign prostatic conditions that can elevate PSA levels, such as BPH or prostatitis. As a result, a substantial proportion of men in this gray zone will undergo unnecessary invasive procedures, such as prostate biopsy, without ultimately being diagnosed with cancer. In the context of prostate cancer screening, particularly for patients with PSA values within the diagnostic gray area, TAP detection exhibits superior diagnostic accuracy, and the combined assessment of TAP and fPSA/tPSA is more efficacious. Although the TAP assay is slightly more expensive than PSA, it remains significantly more cost-effective than magnetic resonance imaging (MRI). In the context of the PSA gray area, the utilization of TAP detection has the potential to curtail superfluous ancillary investigations and attendant expenses, thereby contributing to a more streamlined and economically efficient diagnostic approach.

TAP examination, as simple and cost-effective modality, can be not only used in early diagnosis of cancer but also in post-treatment monitoring of therapeutic efficacy. This could be partially evidenced by existing literature [20]. For prostate cancer, surgery and hormonal therapy are effective and common ways of treatment in the clinic, especially in the early stage which was characterized by satisfying therapeutic effect. Our result proved that TAP expression was lower in patients following treatment compared to pre-treatment levels, indicative of TAP’s sensitivity in tracking treatment responses. It is pertinent to acknowledge that the present study had certain limitations. The study cohort was modest in size, and the follow-up period was relatively brief. Consequently, our investigation should be regarded as an initial appraisal of TAP’s utility in monitoring prostate cancer patients. To substantiate our results, a larger-scale, prospective, multicenter study is warranted.

In conclusion, for patients with prostate cancer, it is extremely crucial to detect cancer at the very early stage and treat disease as soon as possible. Our findings indicate that TAP holds promise as a potential biomarker for prostate cancer due to its enhanced sensitivity, prognostic value, and complementary role to established markers. However, challenges related to standardization, interference factors, cost, and the need for further research must be addressed to fully harness its potential in clinical practice. And, more extensive studies are in great demand to elucidate the potential role of TAP in prostate cancer.

## Data Availability

The datasets used and analyzed in the current study are available from the corresponding authors on reasonable request.
